# Progressive Hepatic Cirrhosis Early After Allogeneic Hematopoietic Stem Cell Transplantation in a Patient with Chronic Hepatitis C Infection

**DOI:** 10.4274/tjh.galenos.2019.2018.0224

**Published:** 2019-05-03

**Authors:** Satoshi Kaito, Noriko Doki, Tsunekazu Hishima, Yasunobu Takaki, Kazuteru Ohashi

**Affiliations:** 1 Tokyo Metropolitan Cancer and Infectious Diseases Center, Komagome Hospital, Hematology Division, Tokyo, Japan; 2 Tokyo Metropolitan Cancer and Infectious Diseases Center, Komagome Hospital, Pathology Division, Tokyo, Japan; 3 Tokyo Metropolitan Cancer and Infectious Diseases Center, Komagome Hospital, Radiology Division, Tokyo, Japan

**Keywords:** Hepatitis C virus, Allogeneic hematopoietic stem cell transplantation, Liver cirrhosis, Early posttransplant period, Fibrosing cholestatic hepatitis

## To the Editor,

Hepatitis C virus (HCV)-infected allogeneic hematopoietic stem cell transplantation (allo-HSCT) recipients have a higher incidence of liver cirrhosis over long-term follow-up compared to recipients without HCV infection [1,2]. However, liver dysfunction related to HCV is usually mild in the first 3 months after allo-HSCT [3]. We present the progressive hepatic cirrhosis soon after allo-HSCT in an HCV-infected recipient. The clinical and histopathological features were very similar to fibrosing cholestatic hepatitis (FCH) caused by HCV reactivation.

A 50-year-old woman with myelodysplastic syndrome with excess blasts-1 was admitted to undergo allo-HSCT. The patient had a history of hepatitis C positivity (genotype 2a) for more than 20 years. Liver enzyme levels at admission were slightly elevated (aspartate aminotransferase 57 U/L, alanine aminotransferase 61 U/L, alkaline phosphatase 434 U/L, cholinesterase 115 U/L, total bilirubin (T-Bil) 1.2 mg/dL, and hepatitis C viral load 2.5x10^4^ IU/mL). The serological tests for hepatitis B virus (HBV) and polymerase chain reaction for HBV-DNA were negative. Computed tomography (CT) demonstrated hepatosplenomegaly. Abdominal ultrasonography (US) showed coarse hepatic echostructure over the entire liver with a dull edge, smooth surface, and straight hepatic vein without ascites or any signs of portal hypertension. Liver biopsy was not performed because of thrombocytopenia.

Just before transplantation, no risk factors except for the mild hepatic dysfunction and age were found, the hematopoietic cell transplantation-comorbidity index (HCT-CI) was 1, and the age-adjusted HCT-CI score was 2 [4,5]. Meanwhile, bone marrow examination revealed active disease with 6.7% myeloblasts. Considering the situation, the patient underwent peripheral blood stem cell transplantation from her human leukocyte antigen-identical sibling after myeloablative conditioning with cyclophosphamide (120 mg/kg) and total body irradiation (12 Gy). Considering drug-induced liver dysfunction, we avoided the use of busulfan. Cyclosporine and short-term methotrexate were used for graft-versus-host disease (GVHD) prophylaxis. After neutrophil engraftment, T-Bil was elevated up to 8.3 mg/dL and hepatitis C viral load was noted to have increased to 4.0x106 IU/mL on day 36 after allo-HSCT. Methylprednisolone was started at 1 mg/kg/day on day 36 for acute GVHD, with gradual improvement in liver test results. We performed deliberate observation of the patient with weekly US and monthly CT after allo-HSCT, which revealed progressive liver atrophy accompanied with ascites.

**Figure 1:**
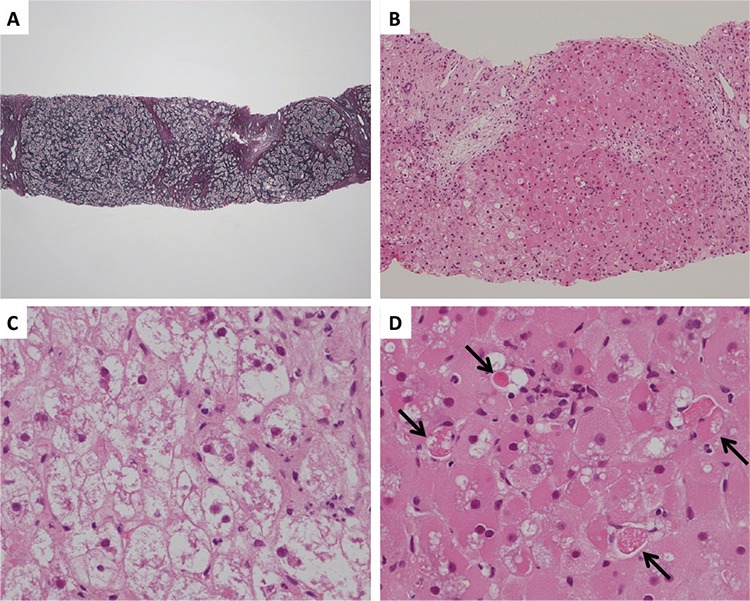
Photomicrographs of transjugular liver biopsy specimen on day 82 after transplantation, when the patient once again became jaundiced and hepatitis C viral load increased. A) There was extensive bridging and pericellular fibrosis with architectural distortion (silver staining, low power field). B) There was severe damage to hepatocytes. Lymphoid infiltration of the portal region was scarce (hematoxylin and eosin staining, low power field). C) Ballooning degeneration of hepatocytes was evident (hematoxylin and eosin staining, high power field). D) The hepatocytes varied in size with oxyphilic and vacuolated cytoplasm. Scattered focal necrosis was evident (black arrow) (hematoxylin and eosin staining, high power field).

**Supplementary Figure 1:**
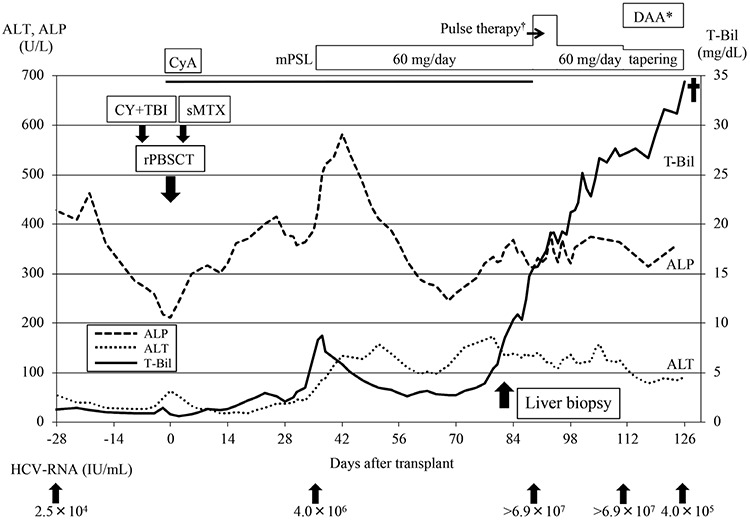
Clinical course of the patient showing serial changes in her liver function. DAA: Direct-acting antiviral therapy, CyA: cyclosporine; mPSL: methylprednisolone, CY: cyclophosphamide, TBI: total body irradiation, rPBSCT: peripheral blood stem cell transplantation from related donor, ALP: alkaline phosphatase, ALT: alanine aminotransferase, T-Bil: total bilirubin, HCV: hepatitis C virus. *DAA included ledipasvir (90 mg/day) and sofosbuvir (400 mg/day). †1000 mg of methylprednisolone was administered for 3 days.

On day 82 after allo-HSCT, the patient once again became jaundiced and hepatitis C viral load increased over 6.9x10^7^ IU/mL. Transjugular liver biopsy showed bridging and pericellular fibrosis with architectural distortion, prominent ballooning, and spotty necrosis, consistent with early cirrhotic changes, and severe hepatocyte damage (Figures 1A-1D). There was mild portal inflammation without histologic evidence of the small bile duct changes of GVHD. Moreover, there was no sinusoidal obstruction. It was unlikely that the hepatopathy would be caused by cyclophosphamide, considering the timing of administration. From the pathological findings and the increased viral load, HCV reactivation was assumed to be the cause of liver dysfunction. Direct-acting antiviral (DAA) therapy with ledipasvir (90 mg/day) and sofosbuvir (400 mg/day) was started on day 110 after allo-HSCT. Although the viral load decreased, the patient developed liver failure and died on day 126 after allo-HSCT (Supplementary Figure 1). 

A few case reports have been published on FCH caused by recurrence of HCV in recipients of liver transplantation [6], renal transplantation [7], and allo-HSCT [8]. The histopathological findings of FCH included periportal fibrosis, ballooning degeneration of hepatocytes, prominent cholestasis, and paucity of inflammation [8]. Although cholestasis was not prominent in our case, other pathological findings and the clinical course were very similar to those of FCH. We speculated that this discrepancy may have been due to the timing of liver biopsy, which was performed immediately after the re-elevation of T-Bil and presumably in the early phase of FCH. 

Generally, the initiation of DAA therapy is recommended at least 3 to 6 months after allo-HSCT in HCV-infected recipients because of the rarity of fulminant hepatitis caused by HCV reactivation in this period and the overlapping toxic effects or potential drug-drug interactions of DAA with other agents [9]. In this case, we started DAA therapy based on the liver pathology and the increased HCV viral load. However, earlier intervention with DAA soon after the initiation of corticosteroid therapy should be considered, because it is a major risk factor for viral replication.

There were some limitations of our clinical practice. First, pretransplant liver status was not fully evaluated. Elastography should be considered for accurate evaluation of the degree of fibrosis [10]. Second, reduced intensity conditioning should be considered to avoid HCV-associated hepatopathy, although in our case the HCT-CI and age-adjusted HCT-CI scores were relatively low. Last, as stated above, earlier diagnosis and intervention with DAA might contribute to good outcomes.

In conclusion, the possibility of HCV recurrence should be also considered as a cause of progressive hepatopathy early after allo-HSCT.

## References

[1] Peffault de Latour R, Lévy V, Asselah T, Marcellin P, Scieux C, Adès L, Traineau R, Devergie A, Ribaud P, Espérou H, Gluckman E, Valla D, Socié G (2004). Long-term outcome of hepatitis C infection after bone marrow transplantation. Blood.

[2] Ljungman P, Locasciulli A, de Soria VG, Békássy AN, Brinch L, Espigado I, Ferrant A, Franklin IM, O’Riordan J, Rovira M, Shaw P, Einsele H;, Infectious Diseases Working Party of the European Group for Blood and Marrow Transplantation (2012). Long-term follow-up of HCV-infected hematopoietic SCT patients and effects of antiviral therapy. Bone Marrow Transplant.

[3] Peffault de Latour R, Ribaud P, Robin M, Valla D, Marcellin P, Socié G, Asselah T (2008). Allogeneic hematopoietic cell transplant in HCV-infected patients. J Hepatol.

[4] Sorror ML, Maris MB, Storb R, Baron F, Sandmaier BM, Maloney DG, Storer B (2005). Hematopoietic cell transplantation (HCT)-specific comorbidity index: a new tool for risk assessment before allogeneic HCT. Blood.

[5] Sorror ML, Storb RF, Sandmaier BM, Maziarz RT, Pulsipher MA, Maris MB, Bhatia S, Ostronoff F, Deeg HJ, Syrjala KL, Estey E, Maloney DG, Appelbaum FR, Martin PJ, Storer BE (2014). Comorbidity-age index: a clinical measure of biologic age before allogeneic hematopoietic cell transplantation. J Clin Oncol.

[6] Satapathy SK, Sclair S, Fiel MI, Del Rio Martin J, Schiano T (2011). Clinical characterization of patients developing histologically-proven fibrosing cholestatic hepatitis C post-liver transplantation. Hepatol Res.

[7] Delladetsima JK, Boletis JN, Makris F, Psichogiou M, Kostakis A, Hatzakis A (1999). Fibrosing cholestatic hepatitis in renal transplant recipients with hepatitis C virus infection. Liver Transpl Surg.

[8] Evans AT, Loeb KR, Shulman HM, Hassan S, Qiu WC, Hockenbery DM, Ioannou GN, Chauncey TR, Gretch DR, McDonald GB (2015). Fibrosing cholestatic hepatitis C after hematopoietic cell transplantation: report of 3 fatal cases. Am J Surg Pathol.

[9] Kyvernitakis A, Mahale P, Popat UR, Jiang Y, Hosry J, Champlin RE, Torres HA (2016). Hepatitis C virus infection in patients undergoing hematopoietic cell transplantation in the era of direct-acting antiviral agents. Biol Blood Marrow Transplant.

[10] Ziol M, Handra-Luca A, Kettaneh A, Christidis C, Mal F, Kazemi F, de Lédinghen V, Marcellin P, Dhumeaux D, Trinchet JC, Beaugrand M (2005). Noninvasive assessment of liver fibrosis by measurement of stiffness in patients with chronic hepatitis C. Hepatology.

